# Xpf suppresses the mutagenic consequences of phagocytosis in *Dictyostelium*

**DOI:** 10.1242/jcs.196337

**Published:** 2016-12-15

**Authors:** Lucas B. Pontel, Judith Langenick, Ivan V. Rosado, Xiao-Yin Zhang, David Traynor, Robert R. Kay, Ketan J. Patel

**Affiliations:** 1 MRC Laboratory of Molecular Biology, Francis Crick Avenue, Cambridge CB2 0QH, UK; 2 Instituto de Biomedicina de Sevilla (IBiS) Hospital Universitario Virgen del Rocío/CSIC/Universidad de Sevilla, Seville 41013, Spain; 3 Department of Haematology, Oxford University Hospitals NHS Foundation Trust, Oxford OX1 2HB, UK; 4 Department of Medicine, Addenbrooke's Hospital, University of Cambridge, Cambridge CB2 2QQ, UK

**Keywords:** Xpf, Phagocytosis, Mutagenesis, *Dictyostelium*, DNA repair

## Abstract

As time passes, mutations accumulate in the genomes of all living organisms. These changes promote genetic diversity, but also precipitate ageing and the initiation of cancer. Food is a common source of mutagens, but little is known about how nutritional factors cause lasting genetic changes in the consuming organism. Here, we describe an unusual genetic interaction between DNA repair in the unicellular amoeba *Dictyostelium discoideum* and its natural bacterial food source. We found that *Dictyostelium* deficient in the DNA repair nuclease Xpf (*xpf*^−^) display a severe and specific growth defect when feeding on bacteria. Despite being proficient in the phagocytosis and digestion of bacteria, over time, *xpf*^−^ *Dictyostelium* feeding on bacteria cease to grow and in many instances die. The Xpf nuclease activity is required for sustained growth using a bacterial food source. Furthermore, the ingestion of this food source leads to a striking accumulation of mutations in the genome of *xpf^−^ Dictyostelium*. This work therefore establishes *Dictyostelium* as a model genetic system to dissect nutritional genotoxicity, providing insight into how phagocytosis can induce mutagenesis and compromise survival fitness.

## Abstract (precis)

**Highlighted Article:** The DNA repair nuclease Xpf helps to maintain the integrity of the genome during bacterial phagocytosis in the amoeba *Dictyostelium*.

## INTRODUCTION

The DNA damage response is highly conserved and prevents the accumulation of deleterious DNA damage after exposure to environmental mutagens. However, organisms and cell types show enormous variability in their susceptibility to mutagen exposure. For instance, in vertebrates, certain lineages such as blood stem cells are highly sensitive to DNA damage, whereas others such as muscle cells are resistant to the same mutagens (Meijne et al., 1991; Rossi et al., 2007). On a quite different scale are organisms such as *Deinoccocus radiodurans* and *Dictyostelium discoideum*, which are highly resistant to mutagens (Deering, 1968; Zahradka et al., 2006; Zhang et al., 2009). It is clear that these organisms have evolved DNA damage responses capable of rapidly and efficiently repairing extensive DNA damage (Hudson et al., 2005). However, an important question remains: why have they evolved such effective DNA repair? One possibility is that both *D. radiodurans* and *Dictyostelium* have unusual life cycles in that they can survive in dormant desiccated states. Such suspended existence could lead to the accumulation of extensive DNA damage that must be repaired to resume growth. Another possibility is that they are exposed to heavy mutagenesis as a consequence of their life cycle or niche, including their food being a source of mutagens. In the wild, *Dictyostelium* feed on bacteria by phagocytosis. The ingested microorganism is trapped in a phagolysosome where it is ultimately killed and degraded in a process resembling that employed by professional phagocytes such as macrophages (Cosson and Soldati, 2008). Here, we show that *Dictyostelium* amoebae use the DNA repair nuclease Xpf to protect their genome from mutagens released during the consumption of bacteria, revealing an unanticipated role of DNA repair in bacterial phagocytosis.

## RESULTS AND DISCUSSION

### A genetic requirement for the DNA repair gene *xpf* to enable *Dictyostelium* to feed on *Klebsiella*

**Fig. 1.**
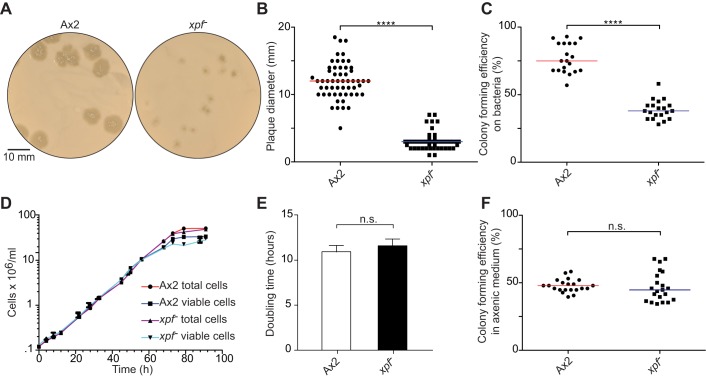
***Dictyostelium* deficient in the DNA repair nuclease Xpf present a growth defect on *Klebsiella aerogenes* lawns but not in axenic medium.** (A) Wild-type (Ax2) and *xpf*-deficient (*xpf^−^*) *Dictyostelium* were plated on agar plates coated with *K. aerogenes* (*K.a*.); single clones of *Dictyostelium* grow out as punched colonies. (B) Quantification of clonal growth of the two strains on *K. aerogenes* plates, scored as plaque diameter at day 5 after growing at 22°C (*n*=55 and *n*=53 for Ax2 and *xpf^−^*, respectively). (C) Colony-forming efficiency of Ax2 and *xpf^−^* on *K. aerogenes* plates (*n*=20). (D) Growth curves for Ax2 and *xpf^−^* in axenic medium. (E) Doubling times calculated from the plot in D (*n*=3, mean±s.e.m.). (F) Colony-forming efficiency in axenic medium (*n*=20). ****P<0.0001; n.s., not significant (*t*-test).

In the wild, the unicellular amoeba *Dictyostelium discoideum* is found in the soil litter feeding predominantly on bacteria and dividing by binary fission (Hohl and Raper, 1963; Weeks and Weijer, 1994). In the laboratory, this organism can be propagated either on agar plates coated with a *Klebsiella aerogenes* bacterial lawn, or it can be grown in axenic medium (Fey et al., 2007). We have previously reported that this organism is highly resistant to the mutagen and DNA-crosslinking agent cisplatin (Zhang et al., 2009). This resistance is under genetic control because *Dictyostelium* deficient in the excision repair nuclease Xpf (*xpf^−^*) are hypersensitive to this DNA-crosslinking agent. However, whenever we propagated the *xpf^−^* strain we noted a profound growth defect on *K. aerogenes* plates (Fig. 1A,B). To address whether this defect was due to a loss of viability, we quantified the colony-forming efficiency. As seen in Fig. 1C, the plating efficiency of Xpf-deficient cells was greatly reduced when grown on *K. aerogenes*. In contrast, this strain grew as well as wild-type (Ax2) *Dictyostelium* in axenic medium (Fig. 1D,E). Furthermore, the cloning efficiency of *xpf^−^* in axenic medium was comparable with that of Ax2 (Fig. 1F), indicating that the vegetative growth defect is specifically associated with feeding on *K. aerogenes* but not with axenic medium. Thus, these data suggest that the *xpf^−^* strain struggles to proliferate when utilizing live *K. aerogenes* as a nutritive source.

### *xpf^−^ Dictyostelium* cells fail to thrive on a range of bacterial strains

**Fig. 2.**
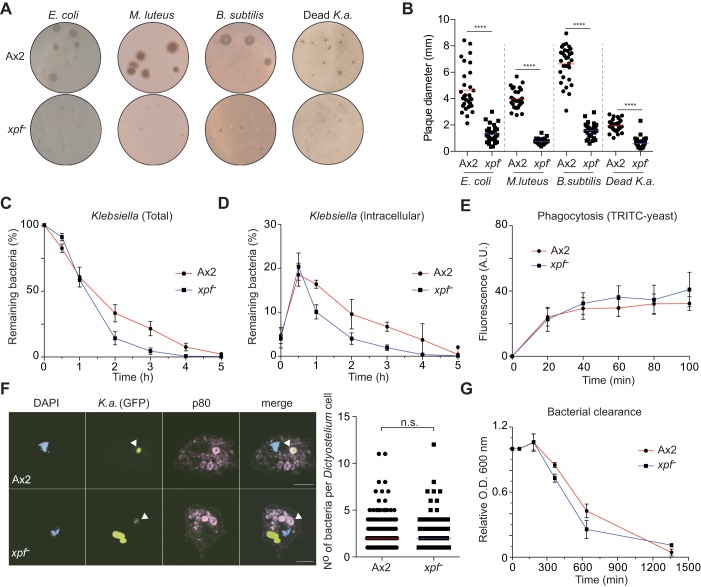
***xpf*^−^*Dictyostelium* are proficient at bacterial phagocytosis.** (A) The *xpf^−^* strain presents a growth defect on *E. coli*, *M. luteus*, *B. subtilis* and heat-inactivated *K. aerogenes* (Dead *K.a*.) plates. (B) Quantification of results in A as plaque diameter (*n*=30). (C) Ax2 and *xpf^−^* strains were incubated with a limiting amount of *K. aerogenes*, and the percentage of remaining live bacilli were then monitored over time (*n*=3, mean±s.e.m.). (D) Similar to C, only that the clearance of phagocytized bacteria was monitored (*n*=3, mean±s.e.m.). (E) Phagocytosis in *Dictyostelium* Ax2 and *xpf^−^* strains scored by incorporation of TRITC-labeled yeast. (F) Uptake of GFP-labeled *K. aerogenes* was monitored by confocal microscopy. White arrowheads indicate the colocalization of *K. aerogenes* with the endocytic marker p80. The number of fluorescent bacteria was quantified within 120 *Dictyostelium* cells. (G) The clearance of bacteria in the supernatant of a suspension containing only *Dictyostelium* and *Klebsiella* was followed by measuring the optical density (O.D.) at 600 nm. ****P<0.0001; n.s., not significant (*t*-test).

We next tested whether this growth defect could be observed with other known bacterial food sources (*Escherichia coli*, *Micrococcus luteus* and *Bacillus subtilis*). *xpf^−^* amoebae showed a growth defect on all of the three quite distinct bacteria to varying degrees (Fig. 2A,B; Fig. S1A). We then whether this effect depended on live food or whether it could also be observed with dead bacteria. We therefore heat-inactivated *K. aerogenes* for 20 min at 121°C. This preparation could still support *Dictyostelium* growth but it is not as nutritious as living *K. aerogenes* (Fig. 2A,B; Fig. S1A). However, heat-inactivated *K. aerogenes* was also toxic to *xpf^−^* amoebae, suggesting that the bacteria do not need to be metabolically active at the time of ingestion to affect *xpf^−^* amoebae.

### Xpf is not required for efficient uptake and digestion of bacteria

In order to feed on bacteria, *Dictyostelium* must internalize their prey and then digest it to release nutrients (Cosson and Soldati, 2008; Lelong et al., 2011). This whole process contrasts to when *Dictyostelium* grows in axenic medium, where nutrient uptake occurs through a process called macropinocytosis (Bloomfield et al., 2015; Cardelli, 2001). We therefore determined whether the *xpf^−^* strain is competent at ingesting and subsequently digesting bacteria. Ax2 and *xpf^−^* strains were pulsed with bacteria for varying times and the number of bacterial colony-forming units (CFU) per amoeba was scored for both the total bacteria bound and internalized by *Dictyostelium* (Lelong et al., 2011). The CFU number did not markedly differ between wild-type and *xpf^−^ Dictyostelium*, strongly indicating that *xpf*^−^ cells do not present a defect in bacterial killing (Fig. 2C,D). We confirmed the bacterial phagocytic proficiency by quantifying the uptake of tetramethylrhodamine isothiocyanate (TRITC)-labeled yeast (Fig. 2E) (Rivero and Maniak, 2006). Finally, when bacteria are ingested by the amoeba, the nascent phagosome incorporates the membrane protein p80 – a reliable marker of the endocytic pathway (Ravanel et al., 2001). Ax2 and *xpf*^−^ strains were therefore pulsed with GFP-expressing *K. aerogenes* and were then visualized by confocal microscopy. Immunofluorescent detection of p80 was colocalized with GFP-containing vesicles. These results show that in both Ax2 and *xpf^−^* cells, GFP-labeled *K. aerogenes* fuse with p80-containing vesicles, causing a decrease in the GFP signal, denoting bacterial digestion (Fig. 2F). Supporting this observation, the clearance in the supernatant of a suspension where both *Dictyostelium* and *K. aerogenes* are co-incubated was similar between the Ax2 and *xpf^−^* strains (Fig. 2G). From this set of experiments, we can conclude that whereas the *xpf^−^* strain is sensitive to *K. aerogenes*, this sensitivity was not due to defective uptake or digestion of bacterial food.

### The endonuclease activity of Xpf is specifically required to tolerate a bacterial food source

**Fig. 3.**
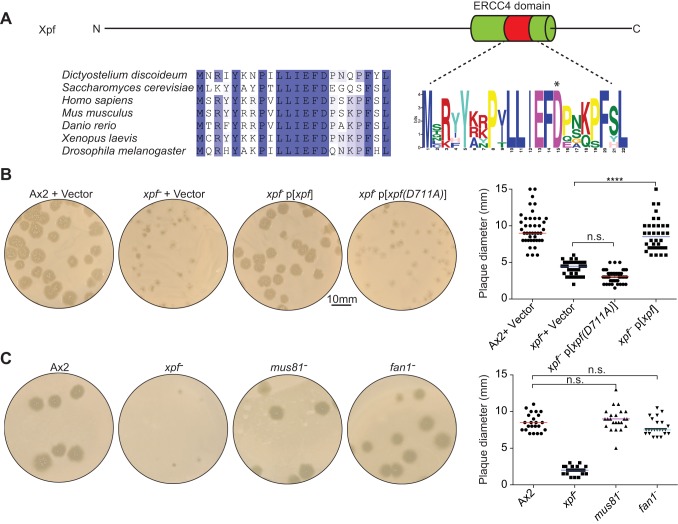
**The nuclease activity of Xpf is required for growth on bacteria.** (A) Domain organization of the Xpf protein; the C-terminal nuclease domain is highlighted to display the high level of conservation and an asterisk marks the crucial aspartic acid residue (D771) that is known to be essential for the nuclease activity. (B) Expression of wild-type Xpf (p[*xpf*]) or the nuclease-inactive point mutant {p[*xpf(D771A)*]; clone 2}. The right-hand panel shows the quantification of plaque diameter at day 5 after growing at 22°C (*n*=42). (C) Growth phenotype on *K. aerogenes* lawns for *Dictyostelium* mutants deficient in other DNA repair nucleases (Mus81 and Fan1) (*n*=23). ****P<0.0001; n.s., not significant (*t*-test).

Xpf is an endonuclease that cuts out damaged DNA caused by UV irradiation and interstrand crosslinking agents (Ahmad et al., 2008; Enzlin and Scharer, 2002). We therefore set out to establish whether this endonuclease activity is required for *Dictyostelium* to effectively utilize bacteria as a food source. Xpf, and in particular its nuclease motif carrying a crucial metal-binding aspartic acid residue, is highly conserved between humans (where the enzyme is also known as ERCC4) and *Dictyostelium* (Fig. 3A) (Enzlin and Scharer, 2002). Consequently, we transfected Ax2 and *xpf^−^ Dictyostelium* cells with plasmids for expression of GFP fusions of either wild-type *Dictyostelium* Xpf (Xpf) or a nuclease-dead mutant form where the key aspartic acid residue in the nuclease motif was mutated to an alanine residue (Xpf-D771A). All transfected strains expressed the recombinant Xpf (Fig. S1B), but only wild-type Xpf and not the nuclease-dead mutant rescued the growth defect of *xpf^−^* cells on *K. aerogenes* bacterial lawns (Fig. 3B; Fig. S1B,C). To extend our analysis, we then tested whether two other DNA repair nuclease-deficient *Dictyostelium* strains [Mus81 (*mus81^−^*) and Fan1 (*fan1^−^*)] were also susceptible to growth inhibition on *K. aerogenes* plates. In fact, neither of these mutants exhibited a growth defect on *K. aerogenes* plates (Fig. 3C). However, *Dictyostelium* has robust DNA repair systems and it has been described as a γ-ray-resistant organism (Deering, 1968; Hudson et al., 2005). Accordingly, we investigated mutants in Xpf-related DNA repair pathways for their contribution to tolerance of bacterial mutagens and found that *Dictyostelium* knockouts in the translesion synthesis DNA-repair polymerase Rev3 (*rev3^–^*) and the global nucleotide excision repair (NER) gene *xpc* (*xpc^−^*) showed a mild growth defect, whereas a *fncD2^−^* strain, which has an inactive Fanconi anaemia DNA repair pathway, showed comparable growth on *K. aerogenes* bacterial lawns to the wild-type strain Ax2 (Fig. S1D). Taken together, our results show that sustained growth on bacterial plates specifically requires the nuclease activity of Xpf.

### Bacterial consumption leads to induced mutagenesis in *xpf^−^ Dictyostelium*

**Fig. 4.**
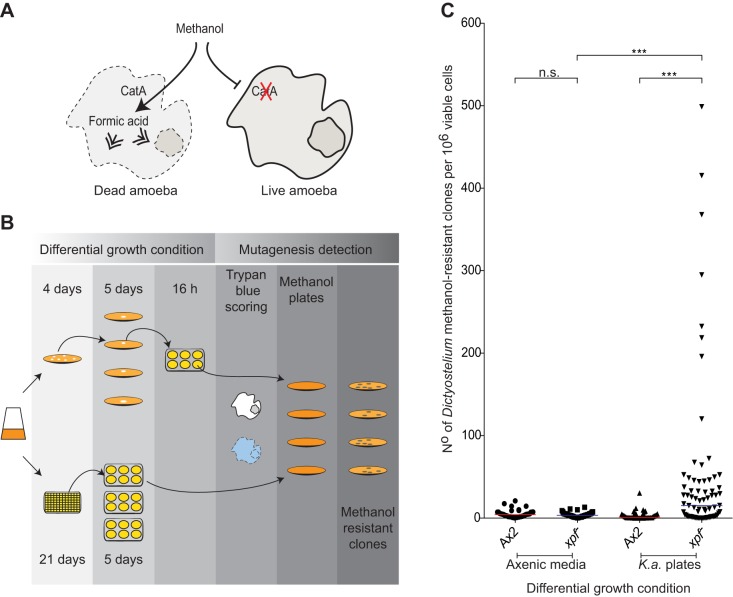
**Consumption of bacteria promotes mutagenesis in *xpf^−^ Dictyostelium*.** (A) Schematic outline of the basis of methanol resistance in *Dictyostelium*. Mutational inactivation of the catalase A gene (CatA) results in failure to break down methanol and hence survival in the presence of this alcohol. (B) Experimental outline of the methanol resistance assay to assess whether growth in axenic medium or on *K. aerogenes* plates promotes the accumulation of *catA* mutations. (C) Graph depicting the number of methanol-resistant clones per 10^6^ viable cells obtained following propagation of Ax2 and *xpf^−^* on either axenic media or *K. aerogenes* (*K.a*.) * *plates. ***P<0.001; n.s., not significant (one-way ANOVA using Tukey–Kramer test for multiple comparison). Each symbol denotes a single clone expanded as shown in B.

The Xpf endonuclease repairs damaged DNA; hence, we reasoned that in its absence, damaged DNA would be expected to accumulate and might eventually lead to mutagenesis. Direct approaches to detect DNA damage were unsuccessful. However, we measured mutagenesis frequency in *Dictyostelium* by assessing their resistance to methanol (Garcia et al., 2002; Podgorski and Deering, 1980). This mutagenic reporter system relies on the fact that methanol is toxic to *Dictyostelium* because of its conversion by the enzyme Catalase A (CatA) into toxic formic acid (Garcia et al., 2002). Inactivation of the *catA* gene leads to a failure to convert methanol into formic acid and hence confers resistance to this alcohol (Fig. 4A). We therefore set up a methanol mutagenesis assay to determine the frequency of resistance to methanol in Ax2 and *xpf^−^* strains after exposure to *K. aerogenes* (Fig. 4B). Briefly, we first took Ax2 and *xpf^−^* cells and expanded them from single clones into six-well plates in axenic medium, and then plated these out on methanol-containing plates. In parallel, we took the same two strains and plated them out onto *K. aerogenes* plates, picked single colonies, and re-plated on new *K. aerogenes* agar plates. We then scraped the entire population from a single plate and briefly expanded them in individual wells within a six-well plate; the expanded population was then plated onto methanol plates. Methanol resistance (as number of resistant colonies per 10^6^ viable cells) was then determined on methanol agar plates. A clone that acquired a *catA* mutation early during growth in any differential condition will show an elevated number of methanol-resistant colonies, but by repeating the assay many times with independent cultures, this fluctuation assay captures the mutation frequency (Luria and Delbruck, 1943). The data in Fig. 4C show that both Ax2 and *xpf^−^ Dictyostelium* developed few methanol-resistant clones when propagated in axenic medium. In contrast, when propagated on *K. aerogenes* plates, the *xpf^−^* strain shows a striking induction of methanol-resistant colonies compared to Ax2, indicating that Xpf prevents mutagenesis in this growth condition. Next, we sought to determine the mutational pattern underlying these mutagenic events and thus amplified, cloned and sequenced the *catA* gene from Ax2 and *xpf^−^* methanol-resistant clones. However, the pattern of mutations did not differ greatly between the two strains (Fig. S2), although it is important to note that the mutational pattern observed here might be biased towards gene-disrupting mutations, which are more likely to cause enzyme inactivation than point mutations.

It is thus very likely that the reason *xpf^−^* amoebae fail to thrive in the presence of a bacterial food source is due to the accumulation of DNA damage. Xpf participates in several DNA repair pathways, including homologous recombination, NER and the Fanconi pathway (Manandhar et al., 2015), which might explain why its role is so fundamental for phagocytic growth. We can only speculate as to the mechanism responsible for causing DNA damage. The simplest explanation is that genotoxins are produced by the ingested bacteria, although it is unlikely to be an exotoxin given that heat-inactivated bacteria were still toxic. It is plausible that the genotoxin might be an integral bacterial part that is not inactivated by heat sterilization, or it could be a substance generated as a consequence of ‘digesting’ this food source. Another intriguing possibility comes from the resemblance of *Dictyostelium* to neutrophils, which kill ingested bacteria by using respiratory burst activity (Chen et al., 2007; Cosson and Soldati, 2008; Zhang et al., 2016). Neutrophil killing generates a battery of reactive molecules such as hypochlorus acid and reactive oxygen species, which are known to be highly mutagenic (Knaapen et al., 2006). It is thus conceivable that the mutagen forms part of an amoebal immune response to bacteria. An intriguing observation is that bacteria differ in their potency to inhibit growth. Exploring which factors determine these differences might give insight into the nature of genotoxicity. Finally, this work highlights the intricate manner by which nutritional sources might stimulate mutagenesis. Consuming food is essential to (heterotrophic) life, but, as this work highlights, often comes at considerable mutational cost against which the organism must defend itself.

## MATERIALS AND METHODS

### Cell culture and molecular biology procedures

All *Dictyostelium* strains were routinely grown at 22°C in HL5 (axenic medium) supplemented with streptomycin (200 µg/ml). Bacterial lawn plates were made by spreading 300 µl of an overnight bacterial culture on SM-agar plates and pictures were taken after 5 days at 22°C. For *Micrococcus luteus*, 0.5 l of stationary-phase culture was pelleted and resuspended in 50 ml, and then 1 ml of this culture was spread on SM-agar plates. Pictures were taken after 9 days. Comparison between two groups was performed by using a *t*-test in Prism software.

The parental strain was the Kay laboratory version of Ax2, according to the following nomenclature: Ax2 (wild type), HM1403 (*xpf^−^*), HM1464 (*mus81^−^*), HM1253 (*fncD2^−^*), HM1456 (*xpc^−^*), HM1351 (*rev3^−^*) (Zhang et al., 2009) and HM1515 (*fan1^−^*; DDB_G0267916). The colony-forming efficiency in HL5 was determined by sorting one cell per well into 96-well plates. After 20 days, the number of confluent wells per plate was scored and represented as a percentage. The colony-forming efficiency on bacterial plates was scored by plating 25 and 50 viable *Dictyostelium* cells on *K. aerogenes* lawns and counting the colonies after 5 days for Ax2, and 6 days for *xpf^−^*. Growth profiles in axenic medium were obtained using a Vi-cell analyzer (Beckman Coulter). Doubling time was determined as described previously (Fey et al., 2007). Cloning of Xpf was carried out using primers described in Table S1 in pDM317. The *Fan1*-knockout was made using a pLPBLP-targeting vector constructed by using the primers listed in Table S1.

### Bacterial killing and phagocytosis assays

Phagocytosis and killing of bacteria were analyzed as described previously (Benghezal et al., 2006). Phagocytosis of fluorescent TRITC (tetramethylrhodamine isothiocyanate)-labeled yeast was based on a published protocol (Rivero and Maniak, 2006). Clearance of bacteria was followed by reading optical density in the supernatant of a phosphate buffer suspension initially containing 10^6^ *Dictyostelium* and 1×10^8^ *K. aerogenes* cells.

### Microscopy

Endocytosis and intracellular bacteria were visualized by incubating 25×10^6^ GFP-expressing *K. aerogenes* from an overnight culture with 5×10^5^ *Dictyostelium* cells in 500 µl of HL5 medium. An anti-p80 H161 monoclonal antibody (2 µg/ml; Pierre Cosson, Department of Cell Physiology and Metabolism, University of Geneva Medical School, Geneva, Switzerland) was used together with an Alexa-Fluor-647-coupled secondary antibody (Mercanti et al., 2006; Ravanel et al., 2001). Images were acquired on a Zeiss LSM710 confocal microscope and processed in ImageJ.

### Methanol sensitivity assay

Ax2 or *xpf^−^* cells were grown in HL5 to 10^6^ cells/ml. From this broth, *Dictyostelium* were plated in order to get isolated clones both in HL5-containing 96-well plates and on *K.-aerogenes-*coated plates. The isolated clones (*n*=41 from HL5, and *n*=43 for Ax2 or *n*=72 for *xpf^−^* from *K. aerogenes* plates) were expanded in HL5. Then, the clonal population was lifted, and up to 5×10^6^ viable amoebae were plated onto 3% methanol-containing SM-agar plates. After 6 days, the number of methanol-resistant colonies per plate was scored and plotted relative to the colony-forming efficiency on *K. aerogenes* plates (Garcia et al., 2002; Podgorski and Deering, 1980). Finally, methanol-resistant clones were grown in HL5 and the *catA* gene was cloned into pTOPO for sequencing.

## References

[JCS196337C1] Ahmad A., Robinson A. R., Duensing A., van Drunen E., Beverloo H. B., Weisberg D. B., Hasty P., Hoeijmakers J. H. J. and Niedernhofer L. J. (2008). ERCC1-XPF endonuclease facilitates DNA double-strand break repair. *Mol. Cell. Biol.* 28, 5082-5092. 10.1128/MCB.00293-08 18541667 PMC2519706

[JCS196337C2] Benghezal M., Fauvarque M.-O., Tournebize R., Froquet R., Marchetti A., Bergeret E., Lardy B., Klein G., Sansonetti P., Charette S. J. et al. (2006). Specific host genes required for the killing of Klebsiella bacteria by phagocytes. *Cell. Microbiol.* 8, 139-148. 10.1111/j.1462-5822.2005.00607.x 16367873

[JCS196337C3] Bloomfield G., Traynor D., Sander S. P., Veltman D. M., Pachebat J. A. and Kay R. R. (2015). Neurofibromin controls macropinocytosis and phagocytosis in Dictyostelium. *Elife* 4, e04940 10.7554/eLife.04940 PMC4374526 25815683

[JCS196337C4] Cardelli J. (2001). Phagocytosis and macropinocytosis in Dictyostelium: phosphoinositide-based processes, biochemically distinct. *Traffic* 2, 311-320. 10.1034/j.1600-0854.2001.002005311.x 11350627

[JCS196337C5] Chen G., Zhuchenko O. and Kuspa A. (2007). Immune-like phagocyte activity in the social amoeba. *Science* 317, 678-681. 10.1126/science.1143991 17673666 PMC3291017

[JCS196337C6] Cosson P. and Soldati T. (2008). Eat, kill or die: when amoeba meets bacteria. *Curr. Opin. Microbiol.* 11, 271-276. 10.1016/j.mib.2008.05.005 18550419

[JCS196337C7] Deering R. A. (1968). Dictyostelium discoideum: a gamma-ray resistant organism. *Science* 162, 1289-1290. 10.1126/science.162.3859.1289 4880786

[JCS196337C8] Enzlin J. H. and Schärer O. D. (2002). The active site of the DNA repair endonuclease XPF-ERCC1 forms a highly conserved nuclease motif. *EMBO J.* 21, 2045-2053. 10.1093/emboj/21.8.2045 11953324 PMC125967

[JCS196337C9] Fey P., Kowal A. S., Gaudet P., Pilcher K. E. and Chisholm R. L. (2007). Protocols for growth and development of Dictyostelium discoideum. *Nat. Protoc.* 2, 1307-1316. 10.1038/nprot.2007.178 17545967

[JCS196337C10] Garcia M. X. U., Roberts C., Alexander H., Stewart A. M., Harwood A., Alexander S. and Insall R. H. (2002). Methanol and acriflavine resistance in Dictyostelium are caused by loss of catalase. *Microbiology* 148, 333-340. 10.1099/00221287-148-1-333 11782526

[JCS196337C11] Hohl H. R. and Raper K. B. (1963). Nutrition of cellular slime molds. I. Growth on living and dead bacteria. *J. Bacteriol.* 85, 191-198. 13961228 10.1128/jb.85.1.191-198.1963 PMC278107

[JCS196337C12] Hudson J. J. R., Hsu D.-W., Guo K., Zhukovskaya N., Liu P.-H., Williams J. G., Pears C. J. and Lakin N. D. (2005). DNA-PKcs-dependent signaling of DNA damage in Dictyostelium discoideum. *Curr. Biol.* 15, 1880-1885. 10.1016/j.cub.2005.09.039 16243037

[JCS196337C13] Knaapen A. M., Gungor N., Schins R. P., Borm P. J. and Van Schooten F. J. (2006). Neutrophils and respiratory tract DNA damage and mutagenesis: a review. *Mutagenesis* 21, 225-236. 10.1093/mutage/gel032 16870698

[JCS196337C14] Lelong E., Marchetti A., Guého A., Lima W. C., Sattler N., Molmeret M., Hagedorn M., Soldati T. and Cosson P. (2011). Role of magnesium and a phagosomal P-type ATPase in intracellular bacterial killing. *Cell. Microbiol.* 13, 246-258. 10.1111/j.1462-5822.2010.01532.x 21040356

[JCS196337C15] Luria S. E. and Delbrück M. (1943). Mutations of bacteria from virus sensitivity to virus resistance. *Genetics* 28, 491-511. 17247100 10.1093/genetics/28.6.491 PMC1209226

[JCS196337C16] Manandhar M., Boulware K. S. and Wood R. D. (2015). The ERCC1 and ERCC4 (XPF) genes and gene products. *Gene* 569, 153-161. 10.1016/j.gene.2015.06.026 26074087 PMC4536074

[JCS196337C17] Meijne E. I., van der Winden-van Groenewegen R. J., Ploemacher R. E., Vos O., David J. A. and Huiskamp R. (1991). The effects of x-irradiation on hematopoietic stem cell compartments in the mouse. *Exp. Hematol.* 19, 617-623. 1893947

[JCS196337C18] Mercanti V., Blanc C., Lefkir Y., Cosson P. and Letourneur F. (2006). Acidic clusters target transmembrane proteins to the contractile vacuole in Dictyostelium cells. *J. Cell Sci.* 119, 837-845. 10.1242/jcs.02808 16478785

[JCS196337C19] Podgorski G. and Deering R. A. (1980). Quantitation of induced mutation in Dictyostelium discoideum: characterization and use of a methanol-resistance mutation assay. *Mutat. Res.* 74, 459-468. 10.1016/0165-1161(80)90176-4 7464851

[JCS196337C20] Ravanel K., de Chassey B., Cornillon S., Benghezal M., Zulianello L., Gebbie L., Letourneur F. and Cosson P. (2001). Membrane sorting in the endocytic and phagocytic pathway of Dictyostelium discoideum. *Eur. J. Cell Biol.* 80, 754-764. 10.1078/0171-9335-00215 11831389

[JCS196337C21] Rivero F. and Maniak M. (2006). Quantitative and microscopic methods for studying the endocytic pathway. *Methods Mol. Biol.* 346, 423-438. 10.1385/1-59745-144-4:423 16957305

[JCS196337C22] Rossi D. J., Bryder D., Seita J., Nussenzweig A., Hoeijmakers J. and Weissman I. L. (2007). Deficiencies in DNA damage repair limit the function of haematopoietic stem cells with age. *Nature* 447, 725-729. 10.1038/nature05862 17554309

[JCS196337C23] Weeks G. and Weijer C. J. (1994). The Dictyostelium cell cycle and its relationship to differentiation. *FEMS Microbiol. Lett.* 124, 123-130. 10.1111/j.1574-6968.1994.tb07274.x 7813881

[JCS196337C24] Zahradka K., Slade D., Bailone A., Sommer S., Averbeck D., Petranovic M., Lindner A. B. and Radman M. (2006). Reassembly of shattered chromosomes in Deinococcus radiodurans. *Nature* 443, 569-573. 10.1038/nature05160 17006450

[JCS196337C25] Zhang X.-Y., Langenick J., Traynor D., Babu M. M., Kay R. R. and Patel K. J. (2009). Xpf and not the Fanconi anaemia proteins or Rev3 accounts for the extreme resistance to cisplatin in Dictyostelium discoideum. *PLoS Genet.* 5, e1000645 10.1371/journal.pgen.1000645 19763158 PMC2730050

[JCS196337C26] Zhang X., Zhuchenko O., Kuspa A. and Soldati T. (2016). Social amoebae trap and kill bacteria by casting DNA nets. *Nat. Commun.* 7, 10938 10.1038/ncomms10938 26927887 PMC4773522

